# HIV and Tuberculosis in Ho Chi Minh City, Vietnam, 1997–2002

**DOI:** 10.3201/eid1309.060774

**Published:** 2007-10

**Authors:** Tran Ngoc Buu, Rein M.G.J. Houben, Hoang Thi Quy, Nguyen Thi Ngoc Lan, Martien W. Borgdorff, Frank G.J. Cobelens

**Affiliations:** * Pham Ngoc Thach TB and Lung Disease Hospital, Ho Chi Minh City, Vietnam; † Academic Medical Center, Amsterdam, the Netherlands; ‡ KNCV Tuberculosis Foundation, The Hague, the Netherlands; § University Medical Center St Radboud, Nijmegen, the Netherlands; 1 Current affiliation: London School of Hygiene and Tropical Medicine, London, UK

**Keywords:** tuberculosis, HIV, Vietnam, epidemiology, substance abuse, injection, trends, research

## Abstract

In Ho Chi Minh City, Vietnam, reporting rates for tuberculosis (TB) are rising in an emerging HIV epidemic. To describe the HIV epidemic among TB patients and quantify its impact on rates of reported TB, we performed a repeated cross-sectional survey from 1997 through 2002 in a randomly selected sample of inner city TB patients. We assessed effect by adjusting TB case reporting rates by the fraction of TB cases attributable to HIV infection. HIV prevalence in TB patients rose exponentially from 1.5% to 9.0% during the study period. Young (<35 years), single, male patients were mostly affected; injection drug use was a potent risk factor. After correction for HIV infection, the trend in TB reporting rates changed from a 1.9% increase to a 0.4% decrease per year. An emerging HIV epidemic, concentrated in young, male, injection drug users, is responsible for increased TB reporting rates in urban Vietnam.

## Abstract 2

An emerging HIV epidemic, concentrated in young, male, injection drug users, is responsible for increasing TB reporting rates in urban Vietnam.

Patients who are co-infected with HIV and *Mycobacterium tuberculosis* are at high risk for active tuberculosis (TB) (*1*). In developing countries, such patients add to the load of already strained TB programs. Although the effect of HIV on the TB epidemic is extensively documented in sub-Saharan Africa (*2*), less is known about its effect in Southeast Asia (*3*,*4*). Although HIV rates in the general Southeast Asian population are still relatively low (*5*), many of these countries have high prevalence of latent TB infection, which makes them vulnerable to the effects of a combined epidemic.

Vietnam is listed by the World Health Organization (WHO) as a TB high-burden country and, through a strong National TB Program (NTP), has reached and exceeded the WHO targets of 70% case detection and 85% cure rates from 1997 onward (*6*,*7*). As a result, TB incidence was expected to decline (*8*), but thus far this has not happened (*9*). One possible explanation for this phenomenon is HIV infection.

The first case of HIV infection in Vietnam was recorded in 1990, and since then HIV infection has been mostly limited to men and high-risk groups such as injection drug users (IDUs) and commercial sex workers (*10*). In more recent years, however, rising HIV infection rates in TB patients have been documented in Ho Chi Minh City, the major urban area with the highest HIV prevalence in the country (*11*). Since 1997, Ho Chi Minh City has also reported increasing TB rates, particularly for young adults (*12*).

Our study objective was to describe the course of the HIV prevalence among TB patients in Ho Chi Minh City during 1997–2002. By combining our data with the NTP reporting data, we also quantified the effect of HIV on the TB reporting rates in this city.

## Methods

### Patient Enrollment

From 1997 through 2002, we performed a repeated cross-sectional survey of HIV prevalence among TB patients in the 12 most urbanized districts (districts 1, 3, 4, 5, 6, 8, 10, 11, Phu nhuan, Tan binh, Nha be, and Binh thanh) of Ho Chi Minh City. Until 1998, districts included all patients >15 years of age who had confirmed TB according to WHO criteria and who consented to HIV testing during the same quarter each year. Since 1999, enrollment was restricted to the last quarter of the year for all districts. Enrollment stopped after a quota was reached; the quota was proportional to the annual number of patients treated in the district, set to obtain a total sample size of ≈800 patients each year. Ethical approval was obtained from the Ho Chi Minh City Council Research Board.

### Measurements

We determined HIV status by ELISA (Genolavia Mixt, Sanofi, Paris, France, until 1999; and Genscreen, Sanofi, Paris, France, from 1999 onward) and an independent confirmatory test (Serodia; Fujirebio, Tokyo, Japan, or Vironostika, Organon, Boxtel, the Netherlands) if the first test result was positive. District TB staff collected data for each patient on TB disease (diagnostic category, treatment history), age, sex, marital and employment status, education level, and risk factors for HIV infection. Patients who owned small businesses and seasonal workers were coded as “self-employed”; civil servants and patients under contract (e.g., drivers), as “employed.” All data were entered twice in EpiInfo version 6 (Centers for Disease Control and Prevention, Atlanta, GA, USA), and discrepancies were checked against the raw data. TB reporting data were obtained from NTP quarterly district reports. Sex, age, and distributions of urban and rural population size were interpolated from the results of the 1994, 1999, and 2004 census; standard exponential population growth was assumed.

### Statistical Analyses

For HIV trend analyses, we used 2-year blocks, increasing group size and power of the analyses. The Cuzick test for trend was used to identify monovariate time trends in HIV prevalence (*13*). To compare proportions, we used the χ^2^ test.

Multivariable analysis was performed by logistic regression. After transformation (squaring), time of inclusion could be entered as a continuous variable (χ^2^ for departure linear trend = 0.68 [df = 1], p = 0.41). Variables were included when the likelihood ratio χ^2^ test was significant at the 0.1 level.

We identified multivariable time trends by entering time interaction variables (time of inclusion × variable x) in our logistic model (*14*,*15*). Compared with the baseline category, an odds ratio (OR) >1 indicates a faster rise in HIV prevalence in that category, thereby identifying high-risk groups. For the sake of interpretability, we present the multivariable model without interaction terms or time trends.

To describe the combined epidemic outside the known high-risk groups, we ran the final multivariable model after excluding all IDUs. The model goodness-of-fit was assessed by the Hosmer-Lemeshow test of goodness-of-fit and by visual inspection of the distribution of the model residuals (*16*,*17*).

We used the formula *p* × (1 – 1/RR) to estimate the fraction of TB in patients that was attributable to HIV (the population-attributable fraction, PAF) (*18*). In this formula, *p* represents the prevalence of HIV in TB patients, and RR represents the relative risk of active TB developing in patients with HIV versus in patients without HIV. The PAF thereby corrects for the fact that some of the TB cases among HIV-infected patients would also have occurred were the patients not HIV infected (*18*). Because this information cannot be known for individual patients, RR represents the average relative risk for HIV-infected patients. Lacking situational data, we conservatively assumed a constant RR of 5 from 1997 through 2002. We based this estimate on the literature and took into account the early stage and specific characteristics (focused in high-risk groups that overlap with TB risk factors such as injection drug use) of the combined epidemic in Southeast Asia (*3*). To test our assumption, the RR was also varied between 2 and 10 or gradually increased (from 2 to 10) over the study period to simulate a progressive fraction of HIV-infected patients in which active TB develops as a result of increased immune suppression.

The rate of TB observed without HIV was calculated as [(1 – PAF) × current TB rate]. We restricted this part of the analysis to new smear-positive TB patients from urban districts. The diagnosis of these patients’ condition is highly standardized and the HIV/TB data came from urbanized districts, which ensured that combining the 2 datasets was as valid as possible. Exponential growth rates were estimated by using the least squares method.

In 2002, the Ho Chi Minh City Council started a mandatory rehabilitation program for IDUs. Because these rehabilitation centers were not included in our surveillance, we quantified potential resulting bias by estimating how including 50% more IDUs in 2002 would affect our results.

Analyses were performed by using Stata version 8 (Stata Corp., College Station, TX, USA). Excel 2003 (Microsoft Corp., Redmond, WA, USA) was used to asses the effect of HIV on reporting rates.

## Results

### HIV in TB Patients

A total of 5,701 patients consented to HIV testing (92% of those eligible) and were included in the study. Because of clerical error, 504 patients entered the study from July through December 1996. These were added to the subset analyzed for 1997. Apart from IDUs, patient numbers in the individual risk categories were too low to be analyzed separately and were therefore added to the category of “other.”

HIV prevalence rose exponentially from 1997 through 2002 (Table 1). The mean age of HIV-infected patients decreased from 38 (standard deviation [SD] = 10) to 27 (SD = 8) years; the mean age of the HIV-negative group remained stable at ≈39 (SD = 14). The male:female ratio was higher for HIV-infected (9:1) than for other TB (7:1) patients (χ^2^ = 53.6 [df = 3], p<0.001). HIV prevalence in young (<35 years) men rose to 22.3% (108/484) during 2001–2002.

**Table 1:** HIV prevalence in tuberculosis patients in Ho Chi Minh City, Vietnam, 1997–2002*

Variable	1997–1998, % (n/N)	1999–2000, % (n/N)	2001–2002, % (n/N)	p value†
Study population	1.5 (38/2,476)	2.9 (47/1,617)	9.0 (144/1,608)	<0.001
Age, y				
<24	0.6 (2/342)	5.9 (14/239)	19.9 (53/267)	<0.001
25–34	2.0 (14/711)	3.1 (14/452)	14.4 (65/450)	<0.001
35–44	2.4 (17/719)	2.4 (11/460)	3.6 (16/439)	0.100
45–54	0.7 (5/704)	1.7 (8/466)	2.2 (10/452)	0.020
Sex				
Male	2.0 (35/1,749)	3.8 (43/1,139)	11.6 (134/1,158)	<0.001
Female	0.4 (3/727)	0.8 (4/478)	2.2 (10/450)	0.001
Marital status				
Married	1.2 (19/1,538)	1.4 (14/989)	4.7 (46/975)	<0.001
Single	1.8 (14/775)	5.4 (28/521)	15.9 (87/549)	<0.001
Separated	3.1 (5/163)	4.7 (5/107)	13.1 (11/84)	0.010
Education level				
Illiterate	1.1 (3/281)	2.0 (3/151)	9.4 (10/106)	0.001
Primary	1.4 (18/1298)	3.8 (18/478)	10.7 (55/512)	<0.001
Secondary or higher	1.9 (17/897)	2.6 (26/988)	8.0 (79/990)	<0.001
Employment status				
Employed	0.9 (3/334)	2.3 (7/300)	5.8 (21/362)	<0.001
Self-employed	1.6 (22/1376)	2.9 (26/909)	8.6 (84/979)	<0.001
Unemployed	1.7 (13/766)	3.4 (14/408)	14.6 (39/267)	<0.001
Risk group				
Injection drug use	31.3 (5/16)	47.1 (8/17)	95.4 (41/43)	<0.001
Other	1.3 (33/2,460)	2.4 (39/1,600)	6.6 (103/1,565)	<0.001
Patient history				
New case	1.6 (33/2,018)	2.9 (39/1,338)	9.5 (129/1,358)	<0.001
Relapsed case	0.5 (1/223)	4.1 (5/122)	5.0 (6/119)	0.002
Other‡	1.7 (4/235)	1.9 (3/157)	6.9 (9/131)	0.030
Tuberculosis type				
Smear-positive	1.5 (27/1,799)	3.3 (38/1,145)	8.3 (91/1,100)	<0.001
Smear-negative	0.3 (1/362)	1.5 (4/260)	5.2 (10/194)	<0.001
Extrapulmonary	3.2 (10/315)	2.4 (5/212)	13.7 (43/314)	<0.001

*%, percentage of HIV-positive patients; n, no. HIV-infected patients; N, total no. patients. †p value for Cuzick nonparametric test for trend across time periods. ‡Includes previously treated tuberculosis patients who did not respond to treatment, defaulted, or received their first treatment outside the National TB Program.

The 12 districts did not differ significantly in HIV prevalence during the study period (χ^2^ = 38.1 [df = 33], p = 0.25) (data not shown). HIV prevalence in reported IDUs rose to 95% in 2001–2002, which accounted for 28% of all HIV-infected patients.

### Multivariable and Time-Trend Analyses

In the multivariable analysis, when time trends and other interactions were disregarded, HIV infection among TB patients was associated with age <45 years, male sex, not being married or employed, and being an IDU (Table 2). As the time-trend analyses show (Table 3), HIV prevalence increased faster in young (<35 years) patients, most prominently in the youngest age group (15–24 years), and in IDUs. Additional interaction (p value for excluding interaction from model = 0.002) between marital status and sex indicated that the high HIV prevalence in single TB patients was mainly attributable to HIV infection among male patients.

**Table 2:** Multivariable model (without interaction terms and time trends) for HIV among tuberculosis patients in Ho Chi Minh City, Vietnam, 1997–2002*

Variable	Crude OR† (95% CI)	p value‡	Adjusted OR§ (95% CI)	p value‡
Year of inclusion	5.78 (4.2–7.9)	<0.001	5.80 (4.1–8.2)	<0.001
Age, y		<0.001		<0.001
<24	6.16 (3.8–9.9)		5.15 (2.9–9.3)	
25–34	4.25 (2.7–6.8)		4.16 (2.5–7.0)	
35–44	1.94 (1.2–3.2)		1.96 (1.1–3.4)	
>45	1		1	
Sex		<0.001		<0.001
Male	5.33 (3.2–8.8)		5.79 (3.4–9.9)	
Female	1		1	
Marital status		<0.001		<0.001
Married	1		1	
Single	3.3 (2.4–4.3)		1.67 (1.2–2.4)	
Separated	2.7 (1.7–4.5)		3.93 (2.2–7.0)	
Employment status		0.18		0.020
Employed	1		1	
Self-Employed	1.31 (0.9–2.0)		1.62 (1.1–2.5)	
Unemployed	1.49 (1.0–2.3)		1.97 (1.2–3.2)	
Risk category		<0.001		<0.001
Injection drug use	76.44 (45.5–128.3)		46.06 (25.3–84.0)	
Other	1		1	

*OR, odds ratio; CI, confidence interval. †Monovariate ORs. ‡p-value for likelihood ratio χ^2^ test for excluding variable from the model. §ORs adjusted for all variables in multivariable model.

**Table 3:** Time trends for HIV among tuberculosis patients in Ho Chi Minh City, Vietnam, 1997–2002*

Variable	OR† (95% CI)	p value‡
Age, y		0.001
<24	4.49 (1.3–15.3)	
25–34	2.82 (0.9–8.6)	
35–44	0.67 (0.2–2.3)	
>44§	1	
Risk category		0.005
IDU	10.56 (1.6–66.6)	
Non-IDU§	1	

*OR, odds ratio; CI, confidence interval; IDU, injection drug user. †OR from time × variable in model; OR>1 indicates a faster rise in HIV prevalence in that category than in the baseline category. ‡p value for likelihood ratio χ^2^ test for excluding variable from model. §Baseline category.

When IDUs were excluded, the multivariable model predicted the data less well (–2 log likelihood with IDUs = –686.7, without = –660.2), but this exclusion affected neither the direction of the ORs nor their size in a relevant way (data not shown). Also, the Hosmer-Lemeshow test of goodness-of-fit remained nonsignificant in both models (p = 0.72 with IDUs, p = 0.69 without). Although IDUs and non-IDU HIV-infected patients did not differ relevantly in age, sex, or marital status (χ^2^ test, p = 0.93, 0.53, and 0.78 respectively), they did differ in their employment status and level of education (χ^2^ test, p = 0.05 and <0.001, respectively).

### TB Reporting Rates

PAF calculations show that 0.7%, 1.5%, 1.3%, 6.7%, 9.5%, and 9.7% of reported new smear-positive TB cases were attributable to HIV in 1997, 1998, 1999, 2000, 2001, and 2002, respectively. After these cases were excluded from analysis, the rising trend in TB reporting rates reversed to a mild decline (Figure, panel A). When stratified for sex (Figure, panel B) and age (Figure, panel C), this effect seemed limited to men and was most prominent in the younger age groups. Increasing the RR over time did not affect the results (data not shown). Adding 50% more IDU patients to the 2002 population enhanced the effect of HIV, which reduced the corrected annual growth in TB reporting from –0.2% to –1.2% in men and from –1.8% to –2.9% in young (<35 years) persons.

**Figure:**
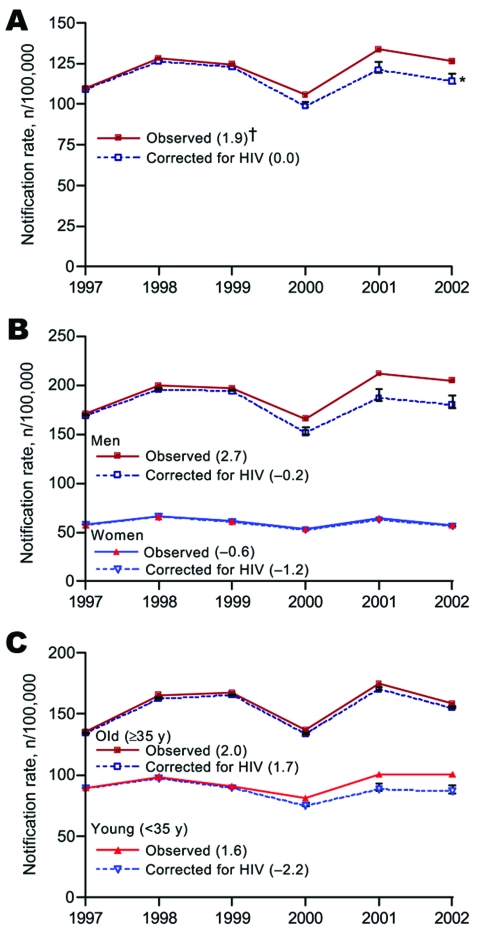
Trends in notification rates of new smear-positive tuberculosis (TB) cases in Ho Chi Minh City, Vietnam, observed and after correction for proportion of cases attributable to HIV infection. Total population (A), sex (B), and age specific (C). Correction of notification rates based on population attributable fraction to HIV infection assuming a risk ratio (RR) of 5 for risk for TB among HIV-infected compared with non–HIV-infected populations. *Error bars indicate corrected rates based on assumption that RR = 2 (top) or RR = 10 (bottom). †Exponential annual change (expressed as percentage) of TB notification rates.

## Discussion

Our results show that Ho Chi Minh City is faced with a combined HIV/TB epidemic that is concentrated and expanding rapidly in young men; injection drug use is a high-risk factor. By 2002, 1 in 10 TB patients was HIV infected, and 1 in 5 men <35 years of age was HIV infected. Even after taking into account the effect of HIV, TB case-reporting rates do not show the decline that is expected if directly observed therapy short course (DOTS) targets are met.

Although the observed trends in HIV infection among TB patients are cause for concern, they are not unexpected. Since 1996, HIV rates have been rising in Vietnam (*10*,*19*). After the average 6-year delay between HIV infection and development of active TB as an opportunistic infection (*20*), HIV infection rates among TB patients were expected to start rising around 2000. Also, the HIV epidemic has mainly affected young men, of whom a large proportion were suspected to have been IDUs (*10*). That this group takes the brunt of the combined epidemic and shows the fastest increase in TB/HIV prevalence is therefore understandable.

The relatively low proportion (28%) of HIV-infected patients who reported injection drug use leaves 72% of HIV-infected TB patients without a clear risk factor. This finding would suggest that HIV has moved beyond the established risk groups and into the general population. However, the strong social stigma associated with injection drug use in Vietnam increases the chance of underreporting; the reported 28% may be lower than actual drug use. The lack of difference in multivariable models with and without reported injection drug use, as well as the similar age, sex, and marital status distributions of IDU and non-IDU HIV-infected patients, supports this possibility.

Under the assumption of a causal relationship between infection with HIV and the risk for active TB (*1*), PAF calculations show that HIV was directly responsible for >9% of TB cases during the last 2 years of our study. This finding explains the increase of TB reporting rates, especially for young men.

The relevance of our data goes beyond the explanation of increasing TB reporting rates in Ho Chi Minh City. Dye et al. predicted that in settings with no HIV, reaching the WHO targets for DOTS would result in an annual decrease of >7% in TB reporting rates (*8*). However, correction for HIV only resulted in a small (0.3%) decline, showing that direct effect of HIV provides only partial explanation for the observed lack of effect of DOTS in Ho Chi Minh City. An additional explanation that we have not studied may be indirect effect of the HIV epidemic, i.e., through increased transmission of *M. tuberculosis* by HIV-infected TB patients. Although recent studies from sub-Saharan Africa have shown mixed results on this issue (*21**–**23*), those data are from settings with a generalized HIV epidemic, and the effect of an epidemic that is concentrated in IDUs may be different, especially in inner city areas.

Other explanations for the lack of decline in TB reporting rates in Ho Chi Minh City include private sector involvement (*24*), internal migration, and perhaps emergence of the Beijing genotype (*25*). In addition, the case detection rate reported by WHO for Vietnam may overestimate that for Ho Chi Minh City. These explanations may apply especially to other parts of Vietnam, where a similar lack of decline in TB reporting rates is observed in the absence of high rates of HIV infection among TB patients (*26*).

### Limitations

Apart from underreporting of risk factors, other limitations may have affected our results. For patients in the districts included in this surveillance, TB may have been diagnosed outside the surveillance project, e.g., in the city’s TB referral hospital or the private sector, which predominantly diagnose smear-negative and extrapulmonary TB. These diagnoses were reported for 29% of the patients in our study compared with 35% of all patients reported in Ho Chi Minh City over the study period. Our data may therefore underrepresent patients with smear-negative and extrapulmonary TB and may have underestimated or overestimated the HIV infection prevalence among them. Our estimates of the impact of HIV infection on TB reporting rates, however, will not be subject to such bias because these were based on new smear-positive patients only.

We have no data on levels of CD4+ lymphocytes and could not stage immune depletion in HIV-infected patients. Whether a case of TB in an HIV-infected patient was due to advanced immune depletion or would have occurred regardless of HIV infection is thus unknown. We have dealt with this possible bias by applying the PAF, which measures excess cases only (*27*). The PAF depends on the RR of TB for HIV-infected persons compared with non–HIV-infected persons and thereby on the level of immune depletion. Because no estimates of this RR are known for the Vietnamese setting, we assumed a value of 5, which is in accordance with RRs found in several studies conducted elsewhere (*3*,*28*,*29*). We also applied values of 2 and 10 and increased the RR over the study period, simulating an increasingly vulnerable population. Neither affected our results in any relevant way.

The withdrawal of IDUs from regular surveillance in 2002 may also have caused bias. But as our simulations showed, the absence of 50% of IDUs reduced the size of the effect but not its direction.

### Recommendations

We recommend that in Ho Chi Minh City all TB patients be tested for HIV because detection of HIV infections can help prevent some of the excess deaths in this population (cotrimoxazole preventive treatment and antiretroviral therapy) (*30**,**31*). To prevent active TB, prophylactic isoniazid treatment for HIV-infected patients could be considered (*31**,**32*). However, a recent study from Ho Chi Minh City showed that isoniazid resistance levels might be too high (>25% in new TB patients) for successful implementation (*33*). Highly active antiretroviral therapy is being introduced and is expected to reduce the risk for TB disease in HIV-infected patients (*34**–**36*). Injection drug use clearly remains a potent source of health problems; efforts to reach out to the vulnerable population of IDUs should be sustained and increased. In addition to the interventions mentioned, TB screening for HIV-infected IDUs and TB treatment for those found to have TB disease should be considered.

## Conclusions

Ho Chi Minh City is now faced with a combined HIV/TB epidemic, predominantly among young men, which reduces the success of TB control. However, HIV alone does not fully explain the lack of a strong decline in TB reporting rates.
